# Transcription factor PbrERF114 is involved in the regulation of ethylene synthesis during pear fruit ripening

**DOI:** 10.1186/s43897-024-00114-2

**Published:** 2024-11-15

**Authors:** Guoming Wang, Zhihua Guo, Tengjiao Wang, Xueping Wang, Kaijie Qi, Jiping Xuan, Chao Gu, Shaoling Zhang

**Affiliations:** 1 Sanya Institute of Nanjing Agricultural University, State Key Laboratory of Crop Genetics & Germplasm Enhancement and Utilization, Nanjing Agricultural University, Nanjing, 210095 China; 2 Jiangsu Engineering Research Center for Germplasm Innovation and Utilization of Pecan, Jiangsu Key Laboratory for the Research and Utilization of Plant Resources, Institute of Botany, Jiangsu Province and Chinese Academy of Sciences, Nanjing, 210014 China

**Keywords:** Pear, Ethylene transcription factor, ACS, Ethylene, Fruit ripening

## Abstract

The plant hormone ethylene is indispensable to the ripening of climacteric fruits. Although extensive studies have been conducted on ethylene signaling, the ethylene response factor (ERF)-mediated transcriptional regulation of ethylene biosynthesis in pear fruits remains to be fully elucidated. We here constructed, sequenced, and analyzed transcriptome libraries in ethephon-treated and 1-MCP-treated Cuiguan pear fruits. In total, 721 fruit ripening-associated differentially expressed genes were identified. Among them, two key genes exhibited positive correlations: the 1-aminocyclopropane-1-carboxylic acid synthase (ACS)-encoding gene *PbrACS3* and the ERF-encoding gene named *PbrERF114*. *PbrERF114* overexpression increased ethylene production as well as the *PbrACS3* expression level. Conversely, virus-induced gene silencing downregulated *PbrERF114*, thereby decreasing ethylene production and reducing *PbrACS3* expression levels. Notably, PbrERF114 could directly interact with *PbrACS3* and *PbrERF24* promoters, thus inducing their expression. However, it did not result in an enhancement in luciferase activity, which is regulated by the *PbrACS1b* or *PbrACO1* promoter. PbrERF24 could directly bind to *PbrACO1* as well as *PbrACS3* to promote ethylene synthesis. In conclusion, PbrERF114 can directly and indirectly mediate ethylene biosynthesis by transcriptionally regulating *PbrACS3* and *PbrERF24*, respectively, thereby triggering a signaling cascade that induces the expression of both *PbrACS3* and *PbrACO1*.

**Supplementary Information:**

The online version contains supplementary material available at 10.1186/s43897-024-00114-2.

## Core

PbrERF114 positively regulates ethylene production during pear fruit ripening. PbrERF114 directly interacts with *PbrACS3* and *PbrERF24* promoters. PbrERF24 directly binds to *PbrACO1* and *PbrACS3*, thereby promoting ethylene synthesis.

## Gene & accession numbers

Gene accessions and sequences can be found in the pear genome database (http://peargenome.njau.edu.cn/)*. PbrACO1* accession: *Pbr031954.1*, *PbrACS1b* accession: *Pbr032688.1*, *PbrACS3* accession: *Pbr015575.1*, *PbrERF114* accession: *Pbr018333.1*, and *PbrERF24* accession: *Pbr012024.1*.

## Introduction

Ripening represents the final stage in the developmental progression of fruits. It involves substantial alterations in sugar/acid contents, flavor, texture, color, and aroma (Klee and Giovannoni 2011). Fleshy fruits are categorized as climacteric and non-climacteric. Ethylene production and respiration increase during climacteric fruit ripening; however, these changes are absent during non-climacteric fruit ripening (Guo and Ecker 2004). Ethylene, a pivotal signaling molecule, is a crucial player in regulating climacteric fruit ripening. Ethylene biosynthesis and signaling pathways have been extensively investigated across various species, including the climacteric pear fruit (Villalobos-Acuna et al. 2011; Li et al. 2014).

Ethylene biosynthesis begins with the ACC synthase (ACS)-catalyzed transformation of S-adenosylmethionine into 1-aminocyclopropane-1-carboxylic acid (ACC). Subsequently, ACC is oxidized by ACC oxidase (ACO) to produce ethylene (Guo and Ecker 2004). During signal transduction, ethylene is detected by the ethylene receptor (ETR), which leads to the production of constitutive triple response 1 (CTR1) (Clark et al. 1998). CTR1 then moderates the inhibition of the downstream positive regulatory molecule ethylene insensitive 2 (EIN2) (Alonso et al. 1999). Later, EIN2 produces signals, thus positively regulating the downstream transcription factors EIN3 and EIN3-like1 (EIL1) (Solano et al. 1998; Chao et al. 1997). Finally, EIN3/EIL1 activation induces ethylene response factors (ERFs), and these factors then initiate the expression of downstream ethephon-responsive genes (Zhang et al. 2009; Gu et al. 2017).

Ethylene biosynthesis and signal transduction have been extensively investigated. Many crucial *ACS* and *ACO* genes involved in these processes have been discovered in various species, such as tomato (Mata et al. 2018; Liu et al. 2018; Guo et al. 2018), kiwifruit (Yin et al. 2010, 2008), banana (Jourda et al. 2014; Xiao et al. 2013), peach (Mathooko et al. 2001; Wang et al. 2017), apple (Li et al. 2016; Tan et al. 2013), and pear (Li et al. 2014; Hao et al. 2018). Ethylene synthesis is known to be controlled through transcriptional regulation. In tomato, LeCp binding to a TAAAAT sequence and RIN binding to a CArG cis-element in the *LeACS2* promoter transcriptionally regulate *LeACS2* (Matarasso et al. 2005; Ito et al. 2008). The binding of AdNAC6 and AdNAC7 to *AdACS1* and *AdACO1* promoters stimulates their expression, thereby increasing ethylene production in kiwifruit (Wang et al. et al. 2020). MdMYC2 binds to G-box cis-elements in *MdACS1* and *MdACO1* promoters, thereby augmenting their transcription in apple (Li et al. 2017). In pear, PuBZR1 interacts with *PuACO1* and *PuACS1a* promoters (Ji et al. 2021). PbbHLH164 binds to the GCC-box cis-element of *PbACS1b* (Guo et al. 2024), and PbHB.G1 and PbHB.G2.1 bind to the *PbACS1b* promoter (Cao et al. 2024), thus directly regulating *PbACS1b* transcription. These findings indicate the importance of transcriptional regulation in ethylene biosynthesis.

The large gene family of ERF plays essential roles across diverse biological processes and under various stress conditions (Gu et al. 2017). These ERFs are pivotal for the ethylene signaling pathway and regulate the expression of ethylene-responsive genes as a response to environmental changes. Direct binding of ERF proteins to the promoters containing GCC-box or DRE cis-acting elements regulates fruit ripening or prevents developmental changes (Li et al. 2016; Wang et al. 2019; Han et al. 2016). In tomato, LeERF2/TERF2 interacts with the DRE element in the *LeACO3* promoter, which results in fruit ripening through the transcriptional activation of ethylene biosynthesis-associated genes (Zhang et al. 2009). TERF1 binds to GCC-box and DRE elements under osmotic stress (Huang et al. 2004). In peach, PpeERF2 binds to *PpeNCED2*, *PpeNCED3*, and *PpePG1* promoters and represses the expression of *PpNCED2/3* and the cell wall degradation-related gene *PpePG1* (Wang et al. 2019). In banana, MaERF11 binds to *MaACS1* and *MaACO1* promoters and suppresses their transcription, whereas MaERF9 activates *MaACO1* promoter activity (Xiao et al. 2013). In apple, MdERF3 binds to the DRE element of the *MdACS1* promoter to increase *MdACS1* expression. MdERF2 binds to the DRE element of *MdACS1* and *MdERF3* promoters, thereby directly suppressing their transcription and inhibiting fruit ripening (Li et al. 2016). In pear, PuERF2 augments *PuACO1* and *PuACS1a* expression by directly binding to their promoters (Ji et al. 2021). However, limited information is available regarding the specific regulatory roles of ERFs in the ethylene signaling pathway or other mechanisms regulating their target genes in pear.

Being among the most well-known fruits, pear (*Pyrus*) is commonly cultivated worldwide (Wu et al. 2013; Gu et al. 2024). The Cuiguan pear is an early maturing *Pyrus pyrifolia* cultivar. It is a climacteric fruit because it releases ethylene during ripening. Previous studies have identified the ethylene biosynthesis genes *PbrACS1b* and *PbrACO1* (*PbrACO54*) and showed that PbrERF24 improves *PbrACO1* expression by directly binding to its promoter in pear (Hao et al. 2018; Guo et al. 2024; Cao et al. 2024). In the present study, 721 differentially expressed genes (DEGs) associated with fruit ripening were identified. Among these genes, two were positively related: the *ACS*- and *ERF*-encoding genes named *PbrACS3* (Cao et al. 2024) and *PbrERF114*, respectively. Transcriptome sequencing revealed that these two genes were involved in fruit ripening regulation. *PbrERF114* was named after it was compared with *Arabidopsis AtERF114*, which is a positive player in stress resistance (Li et al. 2022). PbrERF24 augments ethylene synthesis by binding to *PbrACO1* and directly binding to *PbrACS3* promoters to stimulate their expression. PbrERF114 directly and indirectly promotes ethylene synthesis by binding to *PbrACS3* and *PbrERF24* promoters, respectively. These findings reveal the mechanism through which ERF proteins control ethylene biosynthesis during the ripening of pear fruits.

## Results

### Influence of fruit ripening following ethephon and 1-MCP treatments

**Fig. 1:**
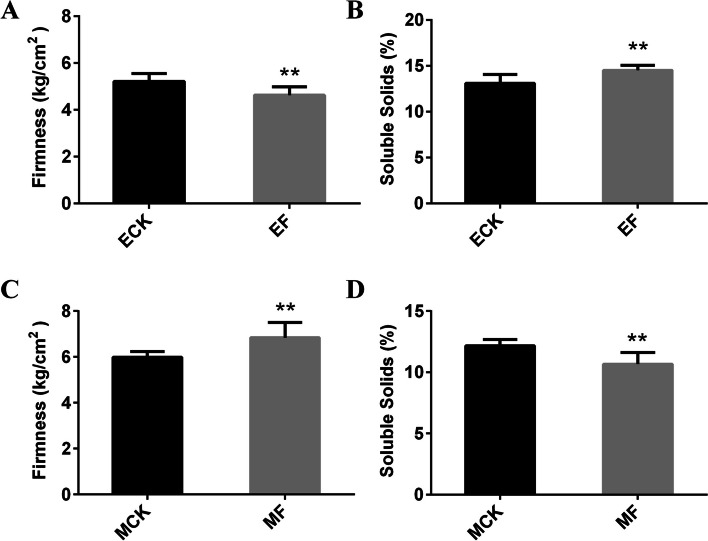
Firmness and soluble solid content of pear fruits treated with ethephon and 1-MCP. Ethephon-treated control fruits (ECK), 300 ppm ethephon-treated fruits (EF), 1-MCP-treated control fruits (MCK), and 1.5 μL/L 1-MCP-treated fruits (MF). At least 10 samples were used for each measurement. Standard errors and analysis of variance were determined using Student’s *t*-test. Single and double asterisks indicate significant differences at P < 0.05 and < 0.01, respectively

Fruit firmness and soluble solid content were used as parameters for evaluating fruit ripeness in sand pear (*P. pyrifolia*). Upon ripening, firmness of sand pears decreases, whereas their soluble solid content increases. Consequently, the firmness and soluble solid content of Cuiguan pear were measured after the application of ethephon and 1-MCP treatments to evaluate fruit ripeness. The ethephon-treated fruit (EF) had significantly lower firmness than the ethephon-treated control fruit (ECK), whereas the soluble solid content was significantly higher in EF than in ECK (Fig. 1). The 1-MCP-treated fruit (MF) had higher firmness than the 1-MCP-treated control fruit (MCK), whereas the soluble solid content was lower in MF than in MCK (Fig. 1). Therefore, EF and MCK were considered mature fruits, whereas ECK and MF were considered immature fruits. These treated samples were analyzed through transcriptome sequencing to isolate pear ripening-related DEGs.

### Identification of genes in response to ethylene

**Fig. 2:**
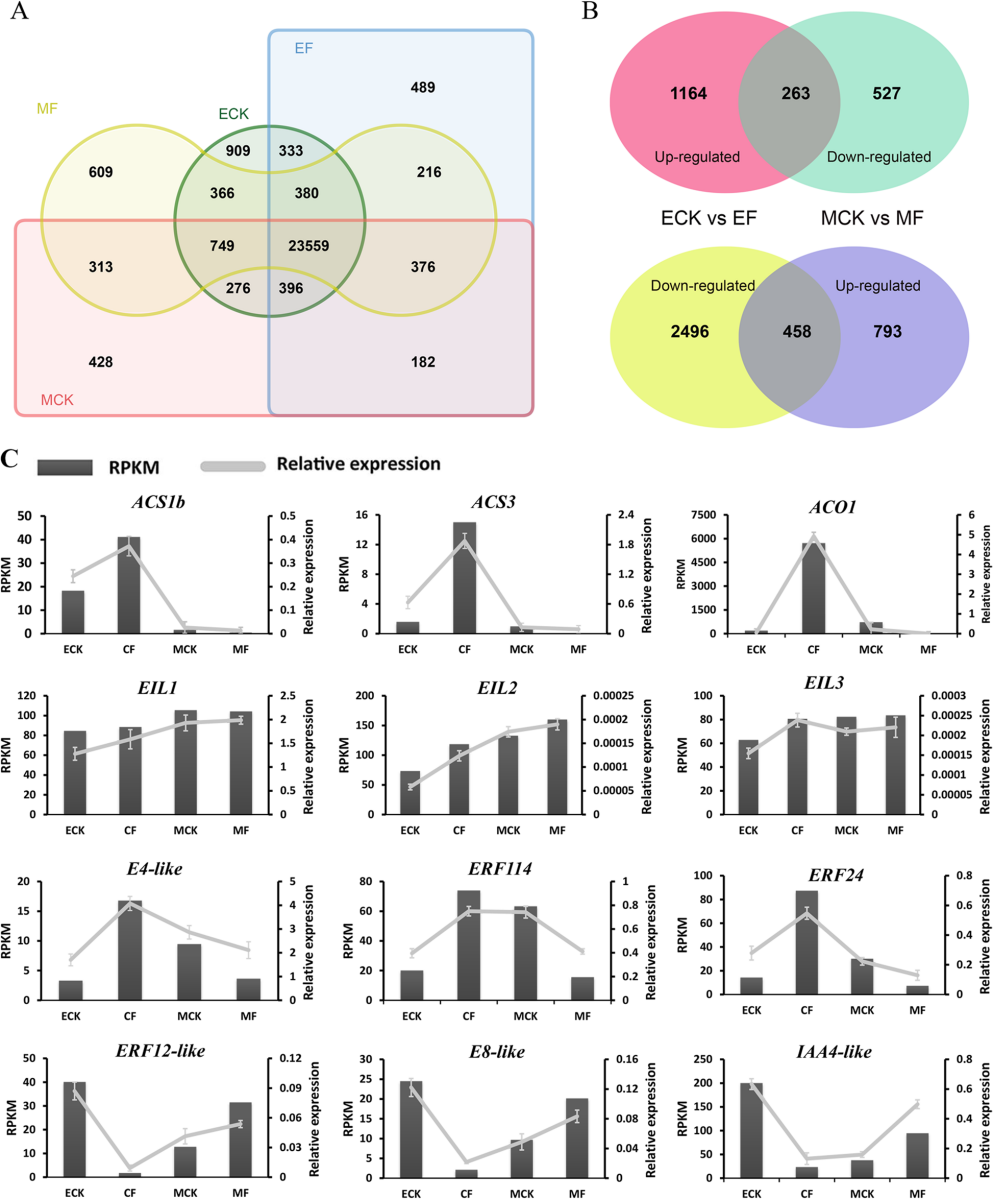
Global and gene expression analyses in ethephon- and 1-MCP-treated pear fruits. **A** Global comparison of ethephon-treated control fruits (ECK), ethephon-treated fruits (EF), 1-MCP-treated control fruits (MCK), and 1-MCP-treated fruits (MF). **B** Potential genes implicated in pear fruit ripening. **C** Validation of expression profiles through qRT-PCR (ECK, EF, MCK, and MF). Three biological replicates were employed for this validation. Standard errors and analysis of variance were determined using Student’s *t*-test

In total, four transcriptome libraries were sequenced from the treated Cuiguan fruit (ECK, EF, MCK, and MF). The adaptor sequences, as well as empty and low-quality reads, were removed, resulting in 21.6, 20.4, 25.5, and 25.7 million clean reads for the ECK, EF, MCK, and MF libraries, respectively (Table S1). Based on uniquely mapped clean reads, 26,968, 25,931, 26,279, and 26,568 expressed genes were identified in the ECK, EF, MCK, and MF libraries, respectively (Fig. 2A). In total, 23,559 genes were commonly identified in all libraries, whereas 909, 489, 428, and 609 genes were exclusively expressed in ECK, EF, MCK, and MF, respectively (Fig. 2A). To identify the DEGs in pear fruits treated with ethephon and 1-MCP, we conducted two pairwise comparisons (ECK vs. EF and MCK vs. MF). In total, 4,381 DEGs were identified between ECK and EF, of which 1,427 genes were upregulated and 2,954 were downregulated (Fig. 2B, Table S3). In the MCK vs. MF comparison, 2,040 DEGs were identified, of which 1,251 genes were upregulated and 790 were downregulated (Fig. 2B, Table S3). These DEGs may respond to exogenous ethephon and 1-MCP treatments or to physiological and biochemical changes occurring during fruit ripening. Ethephon stimulates fruit ripening, whereas the ethylene inhibitor 1-MCP suppresses ethylene signal transduction, thus delaying ripening. Therefore, EF and MCK were considered mature fruits, whereas ECK and MF were considered immature fruits. In total, 263 upregulated DEGs were identified during fruit ripening when upregulated genes from ECK vs. EF were compared with downregulated genes from MCK vs. MF (Fig. 2B, Table S3). Additionally, when downregulated genes from ECK vs. EF were compared with upregulated genes from MCK vs. MF, 458 downregulated DEGs were identified during fruit ripening (Fig. 2B, Table S3).

qRT-PCR was performed to validate the expression levels of candidate genes (Fig. 2C). Notably, we identified four upregulated candidate DEGs involved in the ethylene signaling pathway during fruit ripening: two *ACS* genes (*PbrACS1b* and *PbrACS3*), one *ACO* gene (*PbrACO1*) (Hao et al. 2018; Cao et al. 2024), and two *ERF* genes (*PbrERF114* and *PbrERF24*) (Hao et al. 2018). According to this result, *PbrACS3* and *PbrERF114* genes likely participated in the regulation of fruit ripening. However, three downregulated *EBF* genes (*EBF1*, *EBF2*, and *EBF3*) (Wang et al. 2023) were identified only in the 1-MCP treatment group (MCK vs. MF), and five upregulated *ETR* genes (*Pbr002199.1*, *Pbr004323.1*, *Pbr011796.1*, *Pbr022706.1*, and *Pbr023072.1*) were identified only in the ethephon treatment group (ECK vs. EF) (Table S3). Furthermore, these 721 DEGs identified during fruit ripening were annotated by referring to the Gene Ontology (GO) and Kyoto Encyclopedia of Genes and Genomes (KEGG) databases. Accordingly, these DEGs were classified into molecular function (71), cellular component (27), and biological process (113) categories (Table S4). Overall, 76 KEGG pathways were identified as being significantly overrepresented during fruit ripening (Table S5). To verify the reliability of transcriptome gene expression profiles, qRT-PCR was performed on 12 genes by using specific primers (Table S2). Six DEGs each were upregulated in the immature fruit (ECK and MF) and mature fruit (EF and MCK), respectively. These DEGs were *PbrACS1b, PbrACS3*, *PbrACO1*, *PbrERF114*, *PbrERF24*, and an *E4-like* gene. Three DEGs downregulated in the mature fruit (EF and MCK), namely *E8-like*, *ERF12-like*, and *IAA4-like*, were used to verify transcriptome data. Unexpectedly, although ethylene signaling is a key factor for fruit ripening, the key factors of the ethylene pathway, that is, EIL1, EIL2, and EIL3 (Wang et al. 2023) showed no consistent differences in the treated fruits (Fig. 2C). The relative expression trends of all 12 genes were almost consistent with the transcriptome profiles.

### Effect of nucleus-localized PbrERF114 on ethylene biosynthesis in pear fruits

**Fig. 3:**
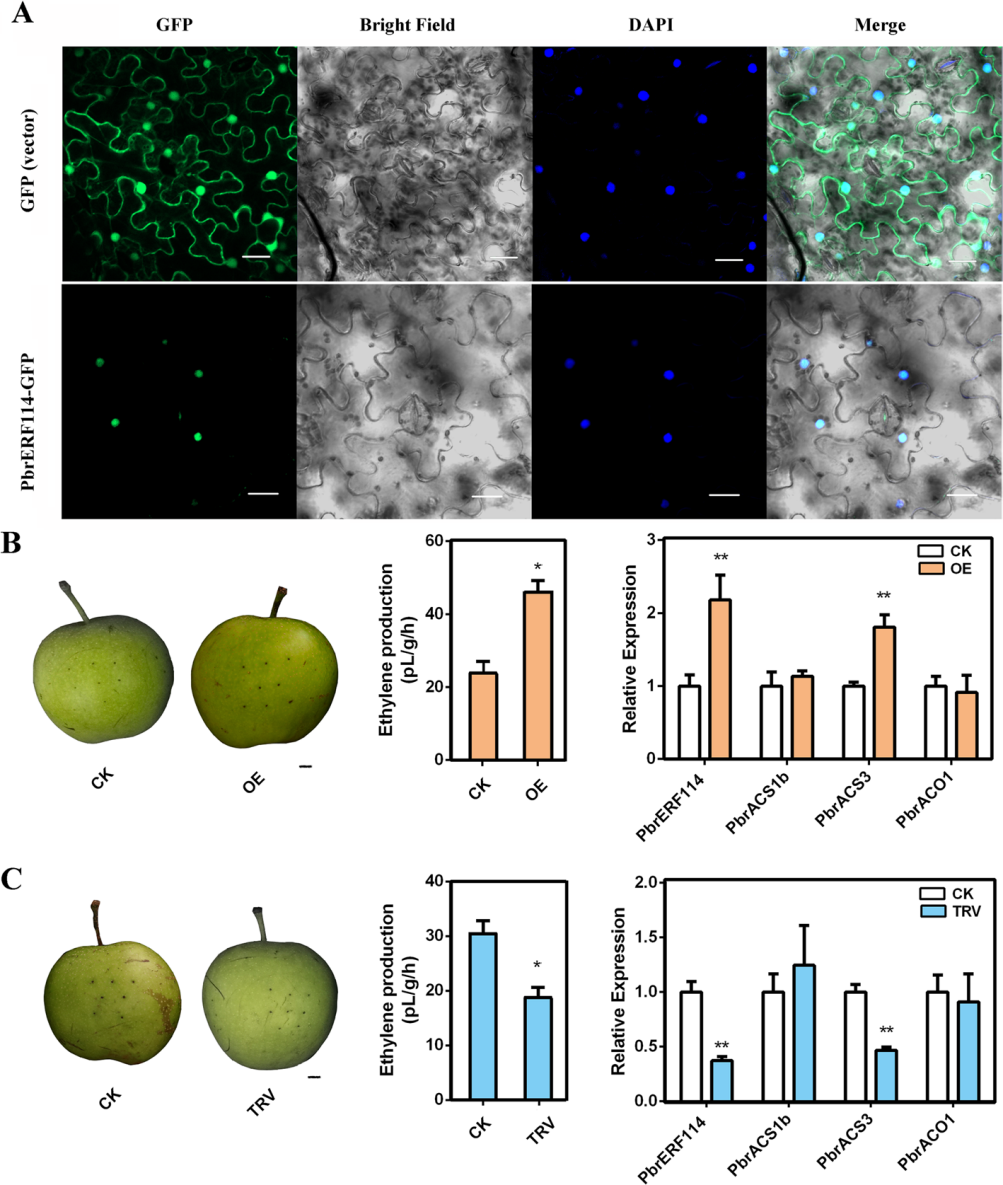
The nucleus-localized PbrERF114 positively regulates ethylene biosynthesis and *PbrACS3* expression. **A** Subcellular localization of PbrERF114. PbrERF114-GFP fusion proteins were localized within the nucleus, as confirmed through DAPI staining. An empty GFP vector served as the control. Scale bar = 20 μm. **B** Fruit flesh transiently overexpressing *PbrERF114* (OE) and the corresponding empty vector control pSAK277 (CK). Scale bar = 1 cm. **C** Fruit flesh with transiently silenced *PbrERF114* (TRV) and the corresponding empty vector control TRV 1 + TRV2 (CK). Scale bar = 1 cm. Six biological replicates were maintained for ethylene production. Three biological replicates are used for validation through qRT-PCR. Standard error and analysis of variance were determined using Student’s *t*-test. Single and double asterisks represent significant differences at P < 0.05 and < 0.001, respectively

To investigate the subcellular localization of the PbrERF114 protein in vivo, a PbrERF114-GFP fusion vector was constructed, and a transient expression assay was conducted using tobacco (*Nicotiana benthamiana*) leaves. The control GFP exhibited a uniform distribution throughout the cell, whereas the PbrERF114-GFP fusion protein was localized within the nucleus (Fig. 3A). This result suggests that PbrERF114 participates in transcriptional regulation, which is consistent with its role as a transcription factor.

To determine whether the putative *PbrERF114* gene responds to ethylene signals for regulating fruit ripening, the overexpression vector pSAK277-PbrERF114, the silencing vector pTRV2-PbrERF114, and pTRV1 were constructed and transiently injected into pear fruit. At 10 days after injection (DAI), ethylene production was significantly higher in the *PbrERF114-*overexpressing fruits than in the control (CK, empty vector pSAK277) (Fig. 3B). Compared with CK, the candidate gene *PbrERF114* and the ethylene biosynthesis-related gene *PbrACS3* were upregulated in the *PbrERF114-*overexpressing fruits (Fig. 3B). Similarly, ethylene production was significantly lower in the VIGS-silenced pear fruit than in CK (empty vectors pTRV2 and pTRV1) (Fig. 3C). Further analysis through qRT-PCR unveiled that both candidate genes, *PbrERF114* and *PbrACS3*, were downregulated compared with that in CK (Fig. 3C). However, *PbrACS1b* and *PbrACO1* exhibited no significant differences in the transgenic fruits with overexpressed or silenced genes. According to our results, *PbrERF114* is among the crucial genes responding to ethylene signals for regulating fruit ripening. Additionally, PbrERF114 regulates *PbrACS3* transcription levels, thereby regulating ethylene production.

### Interaction of PbrERF114 with the *PbrACS3* promoter

**Fig. 4:**
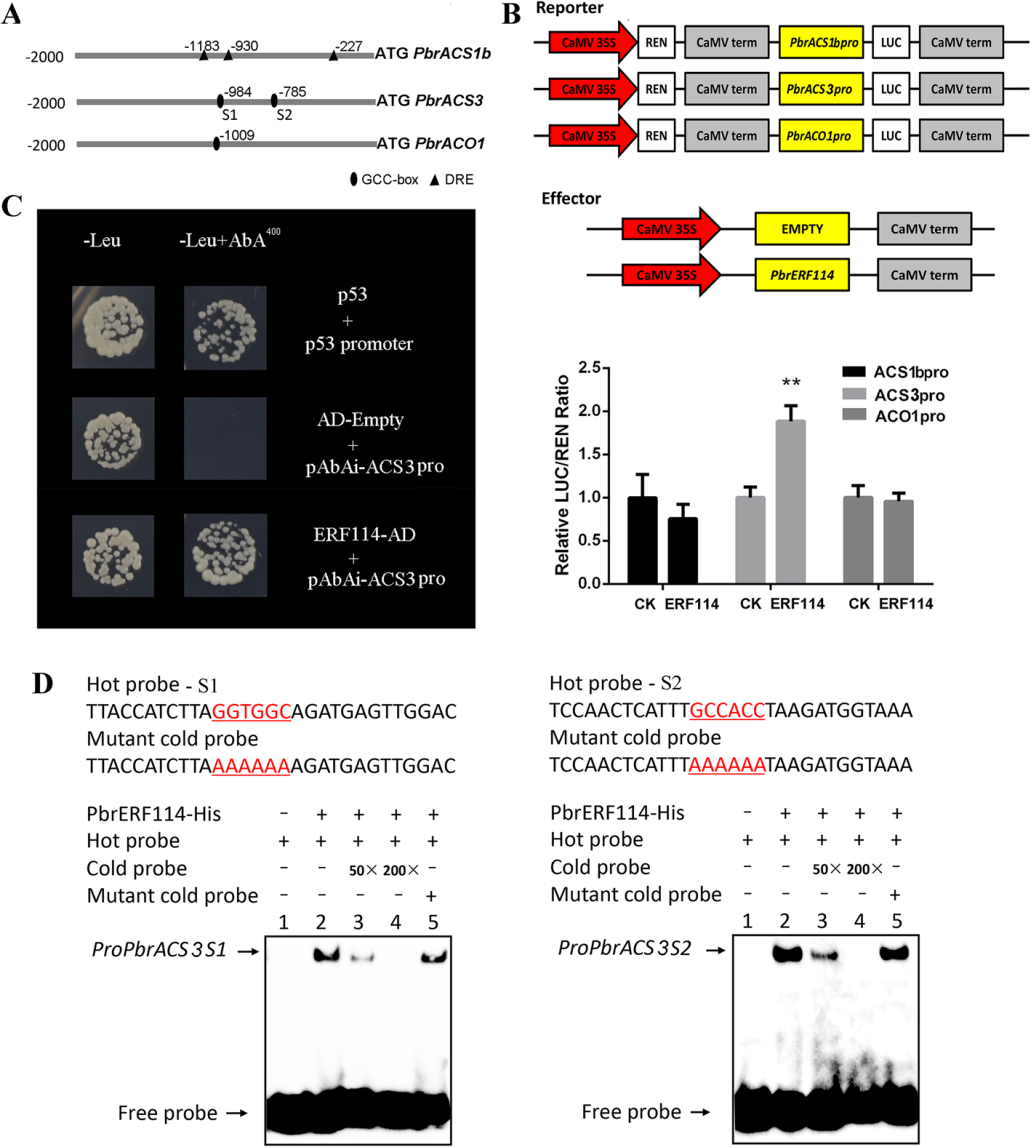
PbrERF114 directly binds to the *PbrACS3* promoter. **A** Predicted GCC-box and DRE elements were identified in *brACS1*, *PbrACS3*, and *PbrACO1* promoters. **B** PbrERF114 induced LUC expression controlled by *PbrACS1*, *PbrACS3*, and *PbrACO1* promoters. Standard errors and analysis of variance were determined using Student’s *t*-test. Single and double asterisks represent significant differences at P < 0.05 and 0.01, respectively. **C** The Y1H assay evaluated the interaction between PbrERF114 and the predicted GCC-box elements in the *PbrACS1* promoter. **D** EMSA examined the binding of *PbrERF114* to the *PbrACS1* promoter. + and − denote the presence and absence of recombinant PbrERF114-HIS, biotin-labeled probe, cold probe, and mutant cold probe, respectively. The cold probe concentrations were tenfold (10 ×), 50-fold (50 ×), and 200-fold (200 ×) those of the labeled probes

ERF proteins directly interact with GCC-box- and/or DREB element-containing promoters, thereby modulating the transcriptional activity of downstream genes and influencing ethylene biosynthesis and signal transduction (Li et al. 2016; Hao et al. 2018; Gu et al. 2017; Zhang et al. 2009). We separately analyzed 2000-bp promoter sequences from three genes, identifying three DRE elements in the *PbrACS1b* promoter, two GCC-box elements in the *PbrACS3* promoter, and one GCC-box element in the *PbrACO1* promoter (Fig. 4A). To investigate the ability of PbrERF114 to modulate the activity of ethylene biosynthesis-related genes, effector and reporter constructs were designed for the dual-LUC assay targeting *PbrACS1b*, *PbrACS3*, and *PbrACO1* promoters (Fig. 4B). The results revealed that LUC activity notably increased when the *PbrACS3* promoter was influenced by *PbrERF114* overexpression, compared with the control empty vector. By contrast, minimal changes were observed in LUC activity, which is associated with the *PbrACS1b* and *PbrACO1* promoters (Fig. 4B). To further confirm the interaction between PbrERF114 and the *PbrACS3* promoter, a Y1H assay was performed. PbrERF114 was found to directly bind to the *PbrACS3* promoter (Fig. 4C). Additionally, electrophoretic mobility shift assays (EMSAs) unveiled the binding of PbrERF114 to *PbrACS3* probes. The binding signal gradually decreased as the cold probe concentrations increased. A similar signal pattern was observed on replacing the cold probe with the mutant cold probe (Fig. 4D). In summary, PbrERF114 directly binds to the *PbrACS3* promoter to augment its activity.

### Interaction of PbrERF114 with the *PbrERF24* promoter

**Fig. 5:**
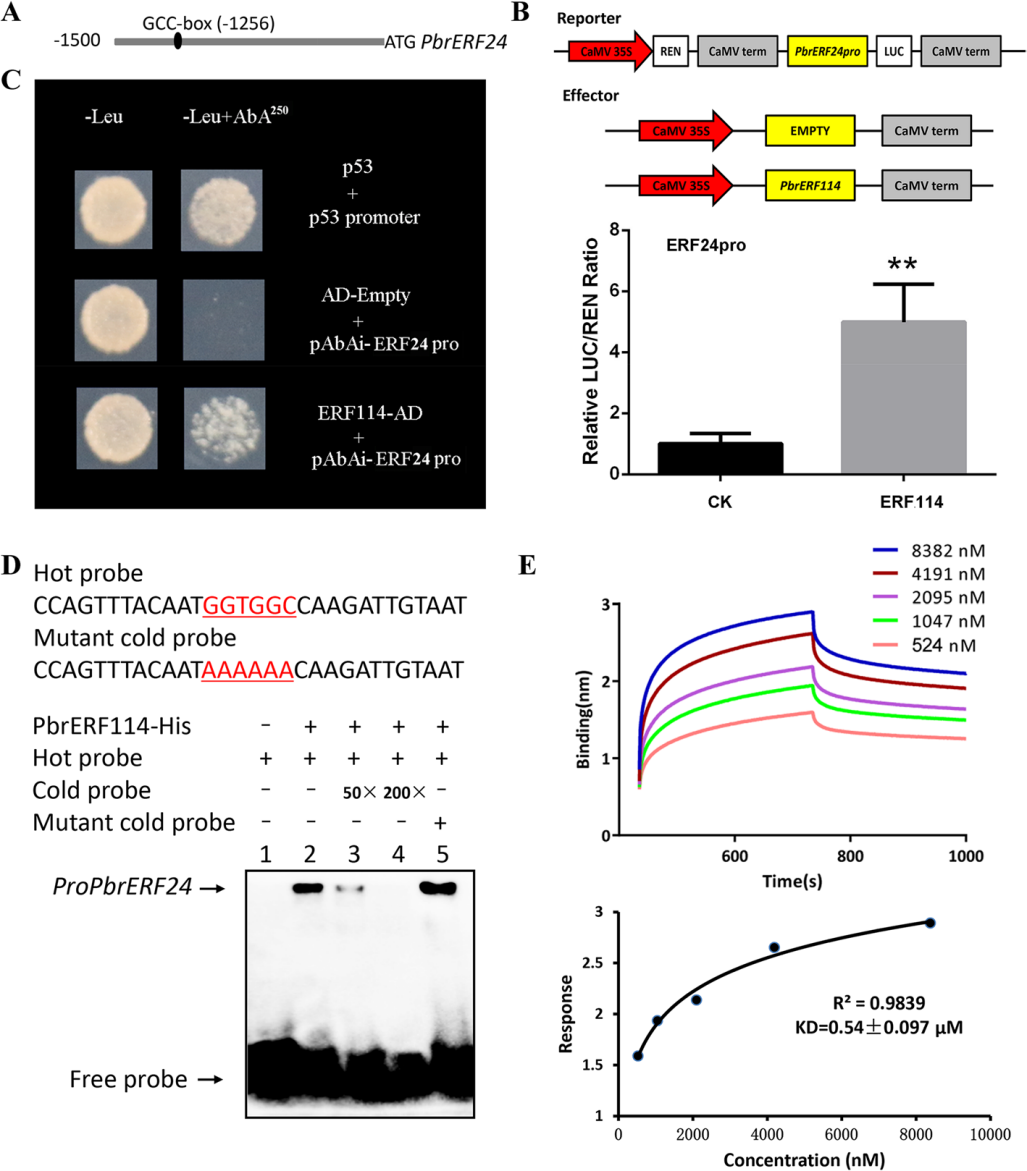
PbrERF114 directly binds to the *PbrERF24* promoter. **A** Predicted GCC-box elements were identified in the *PbrERF24* promoter. **B** PbrERF114 induced LUC expression under the control of *PbrERF24* promoter. Standard errors and analysis of variance were determined using Student’s *t*-test. Single and double asterisks indicate significant differences at P < 0.05 and 0.01, respectively. **C** The Y1H assay assessed the interaction between PbrERF114 and the predicted GCC-box elements in *PbrERF24* promoter. (D) EMSA examined the binding of *PbrERF114* to the *PbrERF24* promoter. + and − denote the presence and absence of recombinant PbrERF114-HIS, biotin-labeled probe, cold probe, and mutant cold probe, respectively. The cold probe concentrations were tenfold (10 ×), 50-fold (50 ×), and 200-fold (200 ×) those of the labeled probes. (E) Kinetic assay of PbrERF114 with the *PbrERF24* promoter

PbrERF24 and PbrERF114 are critical transcription factors in ethylene synthesis as they directly bind to the *PbrACS3* promoter. The GCC-box binding element was identified in the *PbrERF24* promoter sequence (Fig. 5A). Dual-LUC assay was conducted with overexpressed *PbrERF114* and the *PbrERF24* promoter (Fig. 5B). According to the results, PbrERF114 activated *PbrERF24* expression. The Y1H assay confirmed the direct binding of PbrERF114 to the *PbrERF24* promoter (Fig. 5C). Furthermore, EMSAs indicated that PbrERF114-His can bind to the probe. The binding signal decreased as the cold probe concentration increased. A similar signal presentation was observed when the cold probe was replaced by the mutant cold probe (Fig. 5D). In a kinetic assay, the binding affinity between PbrERF114-His and the probe progressively increased as PbrERF114-His concentrations increased. The binding interaction between PbrERF24-His and the probe was saturated at 8382 nM (with a KD of < 1 μM) (Fig. 5E). Taken together, PbrERF114 can directly bind to the *PbrERF24* promoter.

### Binding of the *PbrACS3* promoter by PbrERF24

**Fig. 6:**
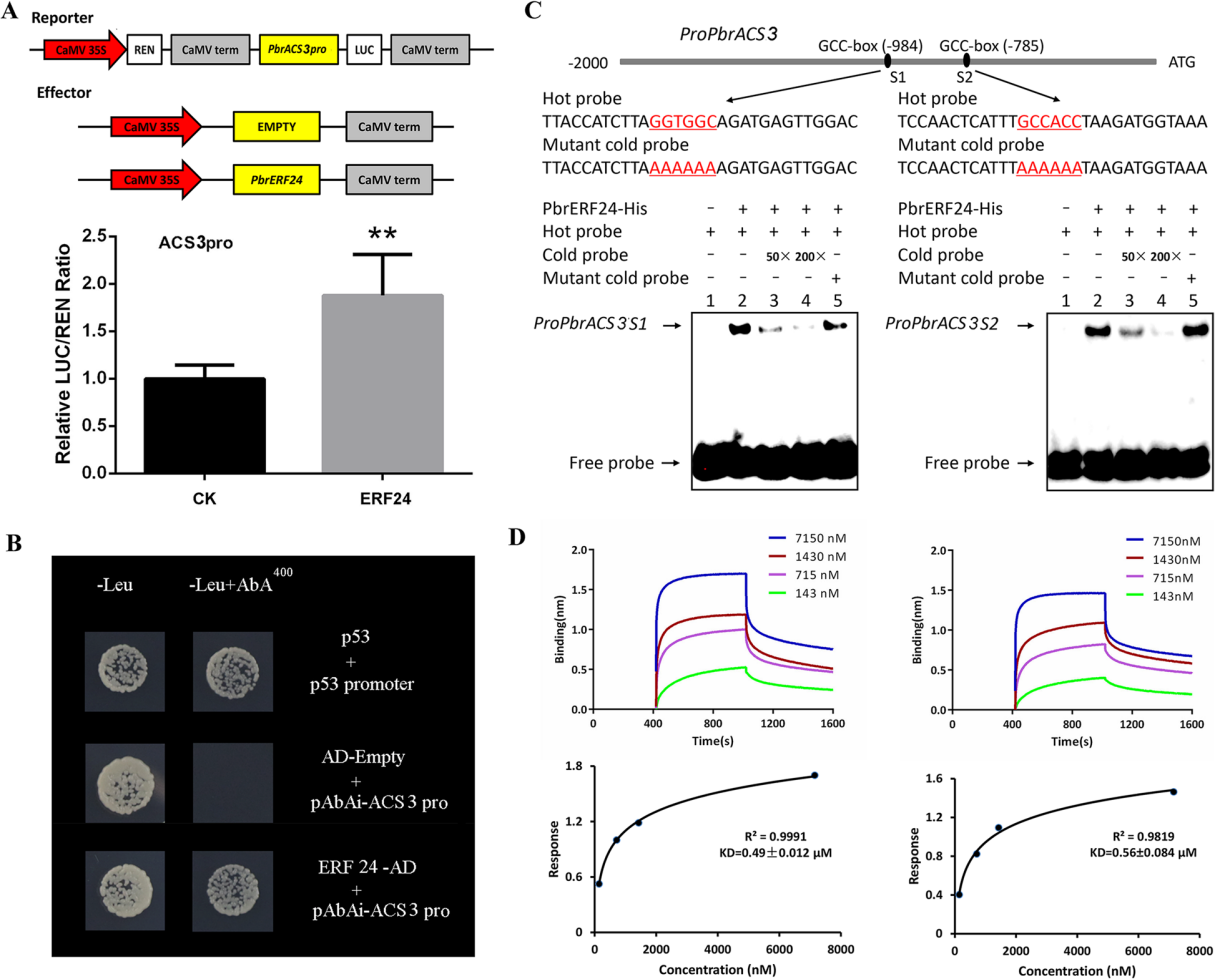
PbrERF24 directly binds to *PbrACS3* promoter. **A** The *PbrACS3* promoter-driven LUC expression was enhanced through PbrERF24 activation. **B** The Y1H assay of PbrERF24 in the *PbrACS3* promoter. **C** EMSA of PbrERF24 with the *PbrACS3* promoter. + and − denote the presence and absence of recombinant PbrERF24-HIS, biotin-labeled probe, cold probe, and mutant cold probe, respectively. The cold probe concentrations were tenfold (10 ×), 50-fold (50 ×), and 200-fold (200 ×) those of the labeled probes. **D** Kinetic assay of PbrERF24 with the *PbrACS3* promoter

We previously reported that PbrERF24 acts as a direct regulator of *PbrACO1* expression by binding to its promoter (Hao et al. 2018; Wang et al. 2023). To assess PbrERF24-mediated potential improvement in *PbrACS3* promoter activity, a dual-LUC assay was conducted, thereby demonstrating its ability to activate *PbrACS3* expression (Fig. 6A). Y1H assays then revealed that PbrERF24 directly binds to the *PbrACS3* promoter (Fig. 6B). Additionally, a recombinant PbrERF24-His fusion protein was generated for EMSA and kinetic assays. EMSA revealed the capability of PbrERF24-His to bind to *PbrACS3* promoter probes, with signals diminishing at higher concentrations of the cold probe. Similar signal patterns were observed following the replacement of the cold probe with the mutant cold probe (Fig. 6C). Furthermore, the kinetic assay demonstrated an increasing binding affinity between PbrERF24-His and the biotin-labeled probe, with a KD affinity constant of < 0.1 μM, which suggested a strong binding affinity (Fig. 6D). Collectively, these findings affirm that PbrERF24 directly interacts with the *PbrACS3* promoter.

### *PbrERF114* is highly expressed during pear fruit ripening

**Fig. 7:**
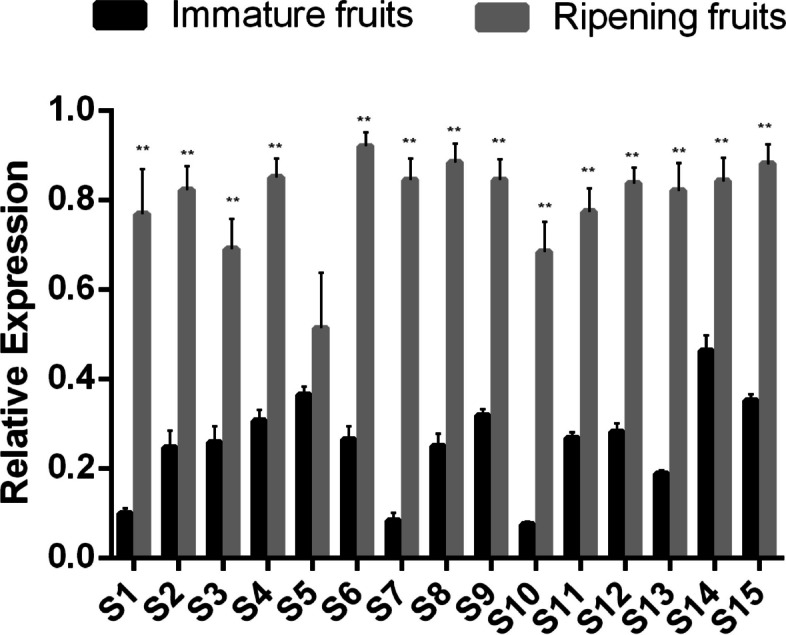
*PbrERF114* is more highly expressed in ripening fruit than in enlarging fruit in different pear cultivars (*P. pyrifolia, Pp; P. bretschneideri, Pb; P. sinkiangensis, Ps;* inter-specific hybridization, *Ph*). The cultivars used were as follows: *Pp* (S1, Nijisseiki; S2, Kisui; S3, Atago; S4, Xueqing; S5, Huanghua; S6, Hangqing), *Pb* (S7, Dangshang; S8, Jinzhui; S9, Yali; S10, Huangguan), *Ps* (S11, Kuerle), and *Ph* (S12, Xizilü; S13, Xinya; S14, Xinhang; S15, Xuefeng)

To determine whether *PbrERF114* expression was involved in immature and mature fruit stages, fruits from 15 additional cultivars were analyzed through qRT-PCR. *PbrERF114* expression was uniformly high in the mature fruits but was extremely low in immature fruits of the 14 pear cultivars (Fig. 7). These results suggested that the *PbrERF114* gene is involved in fruit ripening, thereby supporting its correlation with pear fruit ripening.

## Discussion

Ethylene has been extensively studied as a pivotal hormone influencing the ripening of fruits, particularly climacteric fruits (Liu et al. 2015). In climacteric fruits, *ACS* and *ACO* serve as key genes involved in ethylene synthesis and have been categorized as ethylene response genes (Lee et al. 2011; Li et al. 2016). In pear, *ACS* and *ACO* are involved in ethylene synthesis (Li et al. 2014; Villalobos-Acuna et al. 2011; El-Sharkawy et al. 2004), with limited information available on their molecular regulation. PuBZR1 and PuERF2 bind to *PuACO1* and *PuACS1a* promoters and directly regulate them (Ji et al. 2021). Our previous study identified *PbrACO1* as a key gene involved in ethylene synthesis, which was regulated by ERF24 transcription (Hao et al. 2018). Direct binding of PbbHLH164 to *PbrACS1b* promoter augments its expression (Guo et al. 2024). In *P. ussuriensis, PbrACO1* is similar to the reported *PuACO1*, whereas *PbrACS1b* is different from the reported *PuACS1a* (Li et al. 2014). The Cuiguan pear, an early-ripening cultivar, is a typical climacteric fruit. Transcriptome sequencing of ethephon- and 1-MCP-treated Cuiguan fruits (Fig. 1) resulted in the identification of 721 DEGs involved in fruit ripening, including the ethylene synthesis genes *PbrACS1b*, *PbrACS3*, and *PbrACO1* (Fig. 2). Another DEG isolated, *PbrACS3*, showed similarity to *P. pyrifolia pPPACS2* (BAA76388) and *P. communis Pc-ACS2b* (AAR38503.1), both of which are key *ACS* genes (Itai et al. 1999; El-Sharkawy et al. 2004). However, further screening and verification are required to determine whether additional candidate DEGs, including *E4-like*, *E8-like*, *ERF12-like*, and *IAA4-like* genes, are involved in regulating fruit ripening (Fig. 2).

Fruit ripening has been widely investigated. Some studies have particularly determined the involvement of transcription factors such as RIN (Vrebalov et al. 2002), HD-Zip (Lin et al. 2008), NAC (Ma et al. 2014), ERF (Liu et al. 2018; Li et al. 2016), and bHLH (Guo et al. 2024), which act as activators or repressors, thereby underlining their pivotal roles in climacteric fruit ripening. The *ERF* gene family is the terminal component of the ethylene signaling pathway (Liu et al. 2015; Zhao and Guo 2011). The ethylene signaling pathway and climacteric fruit ripening are controlled by many ERF transcription factors, such as SlERF6 (Lee et al. 2011) and SlERF.B3 (Liu et al. 2018; Liu et al. 2013) in tomato, MdERF2/3 (Li et al. 2016) in apple, MaERF11 (Han et al. 2016) in banana, and AdERF9 in kiwifruit (Yin et al. 2010). Although *ERF* gene family members have been identified in pear (Li et al. 2018), understanding regarding their ability to control the ethylene signaling pathway remain limited. *PuERF2* serves as a DEG during Nanguo pear fruit ripening and regulates fruit ripening by binding to *PuACS1a* and *PuACO1* promoters (Huang et al. 2014; Ji et al. 2021). In our previous study, *PbrERF24* was found to regulate the ripening of Yali pear fruit (Hao et al. 2018). *PbrERF24* overexpression increases ethylene production and *PbrACO1* expression (Hao et al. 2018). Here, based on transcriptome data from various ethephon and 1-MCP treatment groups, the potential involvement of the gene *PbrERF114* in fruit ripening regulation was observed (Figs. 1 and 2). In 14 cultivars, *PbrERF114* expression was higher in mature fruits than in immature fruits (Fig. 3). Moreover, *PbrERF114* overexpression or silencing by transient transformation promoted or suppressed ethylene production, respectively, in pear fruits (Fig. 3), highlighting the role of *PbrERF114* as a positive regulator of ethylene production during fruit ripening.

Many transcription factors, such as EIL (Ma et al. 2020), ERF (Li et al. 2016), NAC (Guo et al. 2021), and bHLH (Guo et al. 2024), transcriptionally activate the ethylene-synthesizing genes, *ACS* and *ACO*, to promote or inhibit ethylene synthesis. According to many studies, the transcriptional activity of ethylene biosynthesis-related transcription factors is influenced by their interacting partners. Identified protein–protein or protein–DNA interactions can enhance or inhibit their impact on *ACS* and *ACO* expression levels. In apple, MdERF2 and MdERF3 proteins interact physically, thereby suppressing MdERF3 binding to MdACS1 (Li et al. 2016). MdERF2 directly binds to the DRE element of the *MdERF3* promoter to suppress *MdACS1* expression (Li et al. 2016). MdMYC2 augments *MdACS1* transcription through the transcriptional control of MdERF3. The MdMYC2–MdERF2 interaction increases *MdACS1* transcription (Li et al. 2017). MdARF5 binds to the *MdERF2* promoter, leading to increased *MdACS3a* and *MdACS1* expression (Yue et al. 2020). PpNAC.A59 activates *PpERF.A16* expression by directly binding to its promoter, which enhances both *PpACS1* and *PpACO1* expression as PpERF.A16 directly interacts with their promoters in peach (Guo et al. 2021). In pear, PuBZR1 indirectly suppresses *PuACO1* and *PuACS1a* transcription by modulating *PuERF2* transcriptional activity (Ji et al. 2021). PbrEILs positively control *PbrERF24* transcription, and PbrERF24 directly binds to *PbrACO1* promoter and enhances its expression (Wang et al. 2023). PbbHLH164 augments *PbACS1b* expression by directly interacting with its promoter, although the interaction with PbRAD23C/D.1 dampens this influence (Guo et al. 2024). In this study, PbrERF114 was found to directly bind to the GCC-box element of the *PbrERF24* promoter to augment ethylene biosynthesis (Fig. 5). However, PbrERF24 does not transcriptionally regulate *PbrERF114*, as revealed through the dual-LUC reporter assay (Fig. S1). Additionally, Y2H unveiled no protein interaction between PbrERF24 and PbrERF114 (Fig. S2). Furthermore, PbrERF24 was found to promote *PbrACS3* and *PbrACO1* expression by directly binding to their promoters (Fig. 6).

**Fig. 8:**
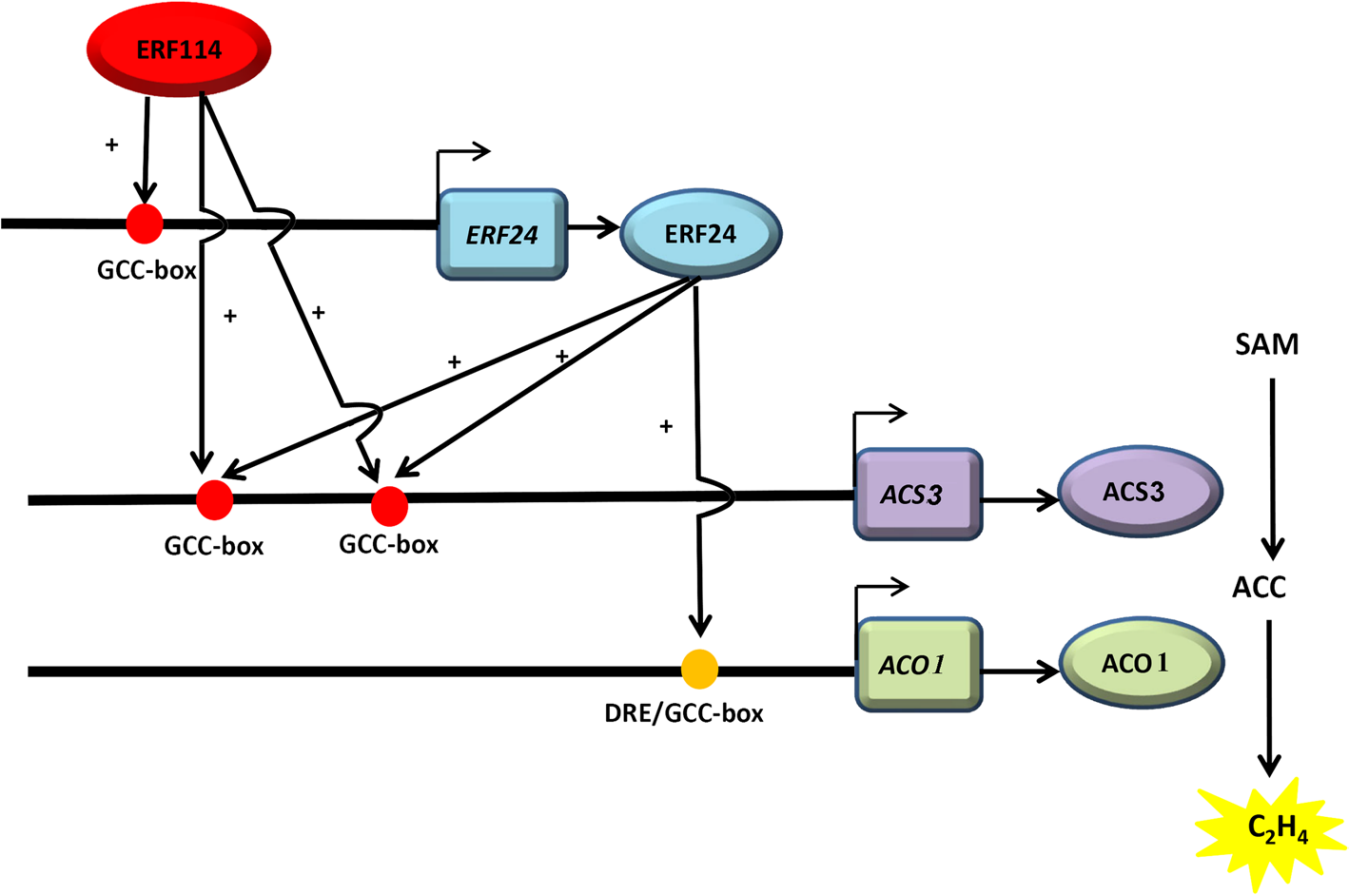
A regulatory model of PbrERF114-mediated ethylene biosynthesis in pear. PbrERF114 activates *PbrERF24* expression by directly binding to its promoter. Additionally, both PbrERF114 and PbrERF24 promote *PbrACS3* expression by directly interacting with their respective promoters. Moreover, PbrERF114 indirectly governs *PbrACO1* expression by regulating the level of PbrERF24, which increases ethylene (C_2_H_4_) production in pear fruit

Taken together, the results indicate that PbrERF114 can directly control ethylene biosynthesis by transcriptionally regulating *PbrACS3* (Fig. 8). Additionally, it can indirectly facilitate ethylene biosynthesis by transcriptionally regulating *PbrERF24*, thereby initiating a signaling cascade that induces *PbrACS3* and *PbrACO1* expression (Fig. 8).

## Materials and methods

### Plant materials and treatment

All Cuiguan pear cultivars used in this study were cultivated at Baima Orchard, Nanjing Agricultural University (Nanjing, China). The fruits were treated with 1000 μL/L ethephon and 1.5 μL/L 1-MCP solutions for 10 min. Approximately 40 fruits from each treatment group were soaked in these solutions at around 30 days before harvest (DBH). Water was used in the control treatment. The treated fruits were enclosed in black airtight bags. These bags were removed after 1 day. Ethephon promotes fruit ripening, whereas 1-MCP inhibits it. When ethephon-treated fruits (EF) ripened, they were considered as samples. On the same day, ethephon-treated control fruits (ECK) were sampled as controls. When the 1-MCP-treated control fruits (MCK) ripened naturally, they were considered as samples. On the same day, samples collected from 1-MCP-treated fruits (MF) considered as samples. The fruits of 15 other cultivars (both 15 DBH immature and 0 DBH mature) of four types were collected: *P. pyrifolia* (*Pp*), *P. bretschneideri* (*Pbr*), *P. sinkiangensis* (*Ps*), and inter-specific hybrids (*Ph*). The cultivars investigated were as follows: *Pp* (S1, Nijisseiki; S2, Kisui; S3, Atago; S4, Xueqing; S5, Huanghua; S6, Hangqing), *Pbr* (S7, Dangshang; S8, Jinzhui; S9, Yali; S10, Huangguan), *Ps* (S11, Kuerle), and *Ph* (S12, Xizilü; S13, Xinya; S14, Xinhang; S15, Xuefeng). All harvested fruits were categorized into three groups. Flesh and peel tissues were separated. The flesh of at least eight fruits were sliced, combined, promptly frozen in liquid nitrogen, and stored at − 80 °C until further analysis.

### Assessment of flesh firmness, soluble solids, and ethylene production

Cuiguan flesh firmness was assessed using a texture analyzer (CT3, Brookfield Engineering Labs. Inc., USA), which was equipped with a 5-mm-diameter probe for penetrating the peeled pulp. Soluble solid content in the fruit juice was measured using a digital hand-held pocket refractometer (PAL-1; Atago, Itabashi-ku, Japan). Referring to the method of Tan et al. (2013), ethylene production was quantified using a gas chromatograph with a flame ionization detector (TRACE GC Ultra—Thermo Fisher Scientific). For each replicate (five replicates), a minimum of three fruits were placed in a 2-L airtight container equipped with septa. The container was maintained at 25 °C. Using a syringe, ethylene gas was sampled from the headspace (five technical replicates) at 4, 6, 8, and 10 DAI. The ethylene production rate was determined using a gas chromatography system (GC2010, Shimadzu, Japan) fitted with a 1000-μL rheodyne injector (Gaoge, Shanghai, China).

### Sequencing and mapping of transcriptome reads

Transcriptome libraries were prepared from the ECK, EF, MCK, and MF samples of Cuiguan. Total RNAs were isolated from these samples by using an RNA kit (Tiangen, Beijing, China). RNA degradation and contamination were visualized on 1% agarose gels. Purified RNA was detected using an Agilent 2100 Bioanalyzer (Santa Clara, CA, USA). Sequencing library construction, PCR product purification, library quality assessment, sample clustering, Illumina sequencing, and clean reads mapping were performed following procedures reported in a previous study (Wang et al. 2022a).

### DEG analysis

Gene expression levels were quantified using reads per kilobase per million (RPKM). The RPKM values were calculated on the basis of gene length and count of clean reads mapped to each gene by using HTseq v. 0.5.4p3. Differential expression analysis was conducted using the DESeq R package V1.18.0 (TNLIST, Beijing, China). Using the Benjamini and Hochberg approach, the false discovery rate and P value were adjusted. DEGs were identified based on P < 0.05 and |log2(fold change)|≥ 1. All DEGs were blasted and annotated according to the NCBI RefSeq nucleotide database, Swiss-Prot and UniProt databases, the Protein family database, GO database, and KEGG ortholog database.

### Quantitative real-time RT-PCR

Total RNA from each sample was separately isolated using an RNA kit (Tiangen, Beijing, China). For reverse transcription, 1 μg RNA was subjected to TransScript®One-Step gDNA Removal and cDNA Synthesis SuperMix (TRANSGEN, Beijing, China). The purified first-strand cDNA samples were used for quantitative real-time RT-PCR (qRT-PCR). Specific primers were designed using Primer Blast (https://www.ncbi.nlm.nih.gov/tools/primerblast/index.cgi). Table S2 lists all primers used. The LightCycler® SYBR Green I Master reagent (Roche, Germany) was used for qPCR. qRT-PCR was conducted using the LightCycler 480 (Roche, Germany). Data were analyzed using the 2^−ΔΔCt^ method (Livak and Schmittgen 2001) with Microsoft Office 2010 software. Pear *PbrTUB* and *Pbr028511* genes were used as internal controls (Wang et al. 2022b). All experiments were conducted with three independent biological replicates.

### Transient transformation in pear fruits

The full-length *PbrERF114* cDNA was amplified from Cuiguan cDNA by using specific primers containing Hpal and XbaI restriction sites. The amplified product was inserted into the binary vector pSAK277, an overexpression vector controlled by the CaMV 35S promoter. Approximately 400-bp specific *PbrERF114* sequences were cloned (Table S2) and inserted into the TRV2 vector. Through heat shock, the pSAK277-PbrERF114 and PbrERF114-TRV2 vectors were introduced into the *Agrobacterium* strain GV3101. These vectors were used for transient transformation in the pear fruits. Previously established protocols were used for the incubation and injection procedures (Hao et al. 2018; Gu et al. 2019). Ethylene measurement and qRT-PCR were performed on transiently transformed pear fruits. All injection experiments were repeated six times.

### Subcellular localization

PCR was performed to amplify the PbrERF114 coding sequence without a stop codon. The C-terminal GFP (pCAMBIA1300-35S)-fused PbrERF114 was constructed in the vector (Xie et al. 2014), with 35S-GFP as the control. Then, 40-day-old tobacco leaves were used for transient gene expression through the *Agrobacterium*-mediated transformation (Sparkes et al. 2006). Two days after transient infiltration, the transformed leaves were treated with 5 μM DIPY for 15 min, and the resulting GFP fluorescence signals were examined through laser scanning confocal microscopy (LSM780, Zeiss, Germany).

### Dual-luciferase assay

As mentioned above, the overexpressing vectors pSAK277-PbrERF114 and pSAK277-PbrERF24 were constructed. These vectors, along with the pSAK277 empty vector, were separately introduced into the *Agrobacterium* strain GV3101. Promoter sequences approximately 2-kb upstream of the initiation codons of *PbrACS1b*, *PbrACS3*, *PbrACO1*, and *PbrERF24* genes were amplified from the genomic DNA of Cuiguan pear (Table S2). The purified promoter fragments were inserted into a pGreenII 0800-LUC vector. The recombinant plasmids produced were transformed into pSoup19-containing GV3101. The transformed *Agrobacterium* solution of pGreenII 0800 was mixed with the *Agrobacterium* solution of pSAK277-35S at a 1:9 volume ratio and injected into 40-day-old tobacco leaves. The empty pSAK277-35S vector served as a negative control. At 3 days after transient infiltration, a luminescence assay of the transformed leaves was performed using the Dual-Luciferase® (Dual-LUC) Reporter Assay System (Promega, Madison, WI, USA) and Cell Imaging Multi-Mode Reader Cytation 3 (BioTek, Santa Barbara, CA, USA). Three independent experiments were conducted, with each comprising three biological replicates.

### Yeast one-hybrid assay

The full-length coding sequences (CDSs) of *PbrERF114* were inserted into the pGADT7 vector, and 2-kb promoter sequences upstream of the *PbrACS3* initiation codon were ligated into the pAbAi vector. The yeast one-hybrid (Y1H) assay was performed using the Matchmaker Gold Yeast One-Hybrid Library Screening System (Clontech, Palo Alto, CA). Table S2 presents all primers used in the study.

### Electrophoretic mobility shift assay

The full-length *PbrERF114* and *PbrERF24* CDSs were inserted into the pCold-TF vector and introduced into* Escherichia coli* BL21. Biotin-labeled probes for PbrACS3 and PbrERF24 were synthesized by Sangon Biotech (Shanghai, China), and the sequences are presented in Figs. 6, 7, and 8. The recombinant proteins and biotin-labeled probes were used for EMSAs. The EMSA was performed using a LightShift Chemiluminescent EMSA kit (Thermo Scientific, Waltham, MA, USA).

### Kinetic assay

Recombinant proteins and fragments of protein-binding elements were obtained as mentioned above. For protein–DNA interactions, biolayer interferometry was performed using a ForteBio Octet 96 Red Instrument (Menlo Park, CA, USA). The kinetic assay proceeded as follows: (1) baseline acquisition was initialized by applying PBS buffer (137 mM NaCl, 4.3 mM Na2HPO4, 2.7 mM KCl, 1.4 mM KH_2_PO_4_, 0.02% (w/v) Tween-20; pH = 7.2) onto streptavidin-coated biosensors, (2) the biotin-labeled probe was loaded onto the sensor, (3) subsequent baseline acquisition was performed using the same PBS buffer, and (4–5) the association and dissociation events between the recombinant protein and biotin-labeled probe(s) were captured. Equilibrium binding affinities (KD) were measured using nanomole sample quantities, whereas the association rate constant (KA) and dissociation rate constant (koff) were assessed to determine the kinetic properties of the binding interaction.

### Yeast two-hybrid assay

In the yeast two-hybrid (Y2H) assay, *PbrERF114* and *PbrERF24* coding sequences were cloned and inserted into the pGADT7 (AD) and pGBKT7 (BK) vectors, respectively. The recombinant plasmids formed were co-transformed into the AH109 yeast strain. The yeast cells were cultured on Trp- and Leu-deficient media (SD/-Trp -Leu) and propagated on a selection medium (SD/-Trp -Leu -His -Ade) to evaluate the interaction for 48–72 h at 28 °C.

## Supplementary Information

Additional file 1: Fig. S1. PbrERF24 does not activate *PbrERF114*, as indicated by the dual-luciferase assay. Fig. S2. PbrERF114 lacks physical interaction with PbrERF24.

Additional file 2: Table S1. Results of transcriptome data and physical map in pear. Table S2. Primer sequences used for cloning, vector construction, and expression analysis. Table S3. List of differentially expressed genes. Table S4. A total of 721 differentially expressed genes analyzed through GO enrichment during fruit ripening. Table S5. 721 Differentially expressed genes analyzed through KEGG enrichment during fruit ripening.

## Data Availability

The datasets used and/or analyzed during the present study are available from the corresponding author on reasonable request.

## Abbreviations

ACC: Aminocyclopropane-1-carboxylic acid
ACO: ACC oxidase
ACS: ACC synthase
DAI: Days after injection
DBH: Days before harvest
DEGs: Differentially expressed genes
Dual-LUC: Dual-luciferase
ECK: Ethephon-treated control
EF: Fruits treated with ethephon
EMSA: Electrophoretic mobility shift assay
ERF: Ethylene response factor
GO: Gene Ontology
KEGG: Kyoto Encyclopedia of Genes and Genomes
MCK: MCP-treated control
MF: 1-MCP-treated fruits
qRT-PCR: Quantitative real-time RT-PCR
RPKM: Reads per kilobase per million
VIGS: Virus-induced gene silencing
Y1H: Yeast one-hybrid
Y2H: Yeast two-hybrid
