# Supplementary material for: Transcription factor PbrERF114 is involved in the regulation of ethylene synthesis during pear fruit ripening
Source: Mol Hortic. 2024 Nov 15;4:38. doi: 10.1186/s43897-024-00114-2 (PMC11566906; doi:10.1186/s43897-024-00114-2)
Supplement: Supplementary file 1 — Additional file 1: Fig. S1. PbrERF24 does not activate PbrERF114, as indicated by the dual-luciferase assay. Fig. S2. PbrERF114 lacks physical interaction with PbrERF24. [file 43897_2024_114_MOESM1_ESM.docx]

**Fig. S1. PbrERF24 does not activate the *PbrERF114* by dual-luciferase assay.**

Six biological replicates were used for the dual-luciferase assay. Standard errors and analysis of variance were determined using Student’s *t*-test.


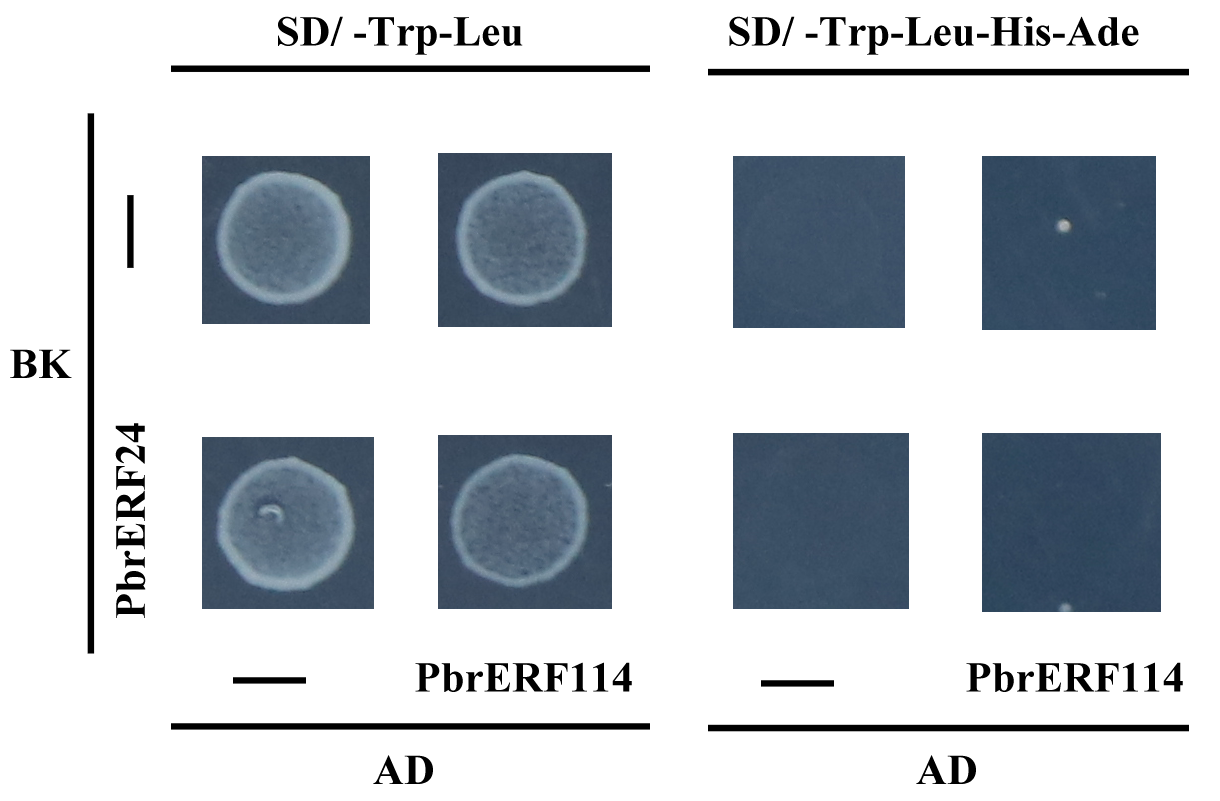
**Fig. S2. PbrERF114 physically no interacts with PbrERF24.**

Yeast two-hybrid assay for *PbrERF114*-AD and *PbrERF24*-BK. Protein-protein interaction was indicated by the ability of cells to grow on synthetic dropout medium lacking Leu, Trp, His and Ade. Yeast grown on 2-dropout medium is the control of the yeast grown on selective 4-dropout medium. The experiment was repeated three times with similar results and representative image is displayed.
